# The Diseases of Society (The Vice and Crime Problem)

**Published:** 1905-08

**Authors:** 


### BOOKS AND AUTHORS.

**The Diseases of Society (The Vice and Crime Problem).** By **G. Frank Lydston,** M.D., Professor of Genitourinary Surgery, State University of Illinois. Small octavo, pp. 626. Illustrated. Philadelphia and London: J. B. Lippincott Co. 1904. (Price, $3.00.)

There is no occasion for surprise in this book when Lydston's meteoric instincts and marvelous versatility are considered, though it must be confessed that one notes, with at least a well-bred lifting of the eyebrows, the radical tone in parts of the argument he advances to strengthen his personal views of some phases of sociologic questions. Generally speaking, the book is well-balanced; and before going into a detailed review it may be set down that the work is important, valuable, and highly instructive.

Lydston has set up a new school of degeneracy and one which will have many followers. Not alone is this so because of his convincing argument, but because even a casual reader cannot help feeling the force and the wisdom of what he says. The book, as a whole, cannot please everyone; and while in the main Lydston's argument is good and his position on certain questions is firm, there are constantly arising doubts as to his sincerity in special departments. We may rail at the laws of nature; we may sympathise with the weak and the dependent, but we cannot hope to revolutionise the laws of man and God by specious argument or the imprint of a single personality, however brilliant, however versatile, however smooth the flow of words.

In his preface Lydston says that he witnessed "a legal murder—the hanging of two unfortunate youths, condemned for an illegal murder." The murder was "unpremeditated;" yet the murderer during a quarrel rushed after a knife and, returning, killed his victim. Certainly, by all the laws of mankind, by all the reasoning of jurisprudence, this was premeditation, in spite of the fact that the youths were "drunk on cheap whiskey, for the drinking of which society itself was indirectly responsible." It is unfortunate that Lydston should have begun his work by what might be considered a damnation of justice as exemplified in this case.

There be those maudlin creatures in this world of ours who would go no further with the book; those self-inflated Spartans who travel on the threadbare and pernicious theory of an eye for an eye and a tooth for a tooth; yet we must not close our eyes to the fact that crime must be punished by the execution of existing laws, and that the Utopian dream of perpetual and promiscuous peace on earth and good will in man is far, far from realisation. Without going into the whys and wherefores of the case, it merely represents Lydston's personally atrophic view of the situation and reflects nothing of the rights and wrongs of existing legal systems.

Personally, the writer, who without egotism, can claim as extensive an experience among criminals as the author of the book, believes in capital punishment. He has witnessed something over a score of hangings, and he believes the world was the better and social conditions improved by the execution of every criminal he has seen die on the scaffold. A fertile field of argument is opened up by Lydston's position on this "legal murder." Society is in a measure responsible for much of the crime of the world, as he says. The rich commit crimes and go unpunished; the poor man steals bread to feed his hunger and is sent to jail. Yet, why should the poor man steal bread when he could have had it for the asking? There's the query of the reformer. The answer is simple, perhaps brutal. He stole because organised charity often compels him to steal,—organised charity which has a flea-bitten fad; which objects to "educating paupers," and which investigates before it feeds. In the gnawing interim the hungry man steals. Because of this he is "unworthy," and from then on God help him unless he falls into the pace of some coarse grained amateur whose heart is full of sympathy and who gives him food without first making inquiry as to how it happens that he is so far fallen from decency as to go about the streets wearing soiled linen when there is water enough in the lake to cleanse unwashed thousands? To that extent is society responsible for fallen conditions.

We must agree with Lydston in his plaint that there has been too much preaching and too little attention paid to the criminal's body. Punishment and preaching are insufficient as reformative measures. The natural atavistic tendency of human nature resents it. In a rather close association with criminal classes the writer knows but one instance where a pledge was broken or a favor forgot when honor was at stake,—and the honor of a thief is dependable; it hasn't the fine finish of the honor of society, but it lies thicker and wears longer in many cases. Punitive, corrective and repressive legislation as a preventive of crime, when taken alone is a failure, as Lydston states; quite as much so as was Brockway's paddling bees at Elmira Reformatory some years since. The body and the mind must be cared for. This accounts for the vast amount of good done by the Salvation Army and the Volunteers of America. Where the society reformer attempts to teach the toil worn wife of the day-laborer with a horde of ill-fed and unkempt children clinging to her skirts, new designs in tatting and pleads the salvation of her immortal soul, the workers in the blue and gray roll up their sleeves, get down on their knees and scrub and clean and lighten the burden of the mother and teach her to make a pleasant home. They feed first and preach afterward.

General Booth early recognised the value of a full stomach as an easy road to a clean life. He anticipated, in a way, Lydston's tenable theory of autointoxication as a factor in the causation of crime. He applied the cure in a homely and common sense, but none the less satisfactory, manner; and it is quite safe to say that for every redemption by the recognised church, there stand hundreds to the credit of the two armies. Lydston does not say this, but the practice of the armies is the formula he presents for the improvement of the criminal classes. They are today doing the work he advocates in the care of the children of the poor; in the uplifting and upbuilding of their minds, and in the nurturing of their bodies; in the fostering of the good that is in them and the crushing out of the elements of the bad.

The professional labor agitator is responsible for the enforced idleness in state prisons spoken of by the author with none too caustic a manner. The kow-towing of the politician to the labor vote made possible the passage of laws snatching from the convict the only happiness he had,—work. There too, is society at fault in its brainless, boneless toddling in the wake of party leaders. It shows a serious study of the criminal by Lydston when he declares that sentiment has no place in the treatment of the criminal. He speaks of fashionable women presenting "bouquets and frosted cake to criminals."

To the lay mind this may sound exaggerated or merely the repetition of newspaper gossip, but it is true. The writer has seen a condemned murderer's cell in the Tombs prison loaded, —literally loaded,—with fruits, flowers and fancy furnishings; books, bouquets and bon-bons; candy, cakes and crosses; while every mail brought him gushing letters from hysteric women and perverted girls. During visiting hours they begged for admission to the sickening disgust of the warden. And the crime for which this murderer was to die was the wanton slaughter of his pregnant sister-in-law because she refused him more money to treat the mixed-ale gang of degenerate toughs of which he was the leader.

Lydston's chapter on the principles of evolution might well be read and printed and reprinted and scattered broadcast. It is a statement of plain truths which sets forth in caustic language the present status of society in a manner, convincing and forceful. So apparent is the truth of what Lydston says that none can challenge his statements. Neuropsychic degeneracy explains much of the crime of the world, while simple degeneracy is responsible for inebriety, insanity, epilepsy, prostitution, and pauperism. Of enthralling interest is the chapter on etiology of social diseases. It is logical and is the basis of a new school of criminology,—a school which will thrive and grow and become the basis of sane and sensible practice, because it is American.

All save the saturated sentimentalist and the purblind, professional parishioner will applaud Lydston for the stand he takes on the slum question. Here, really, is the birthplace of general crime. Here prostitution is flaunted in the eyes of the world and society shudders and give alms for foreign missions,—alms taken from the rentals received from brothels where bastards are begot and where moral crime is a necessity. The moral laxity of the slums made it possible for a 9-year old boy to contract gonorrhea from a 12-year old girl as reported by the writer in the JOURNAL a year or so ago. Lydston lays the blame for such conditions, and very properly, at the doors of society which ignores the children of the slums until they are old enough to commit crime,—and then it jails them.

The press and the cheap theater come in for their share of a just condemnation; the former for invading private lives, the latter for its pernicious, red-fire type of nasty melodrama. No newspaper dare print the truth about the cheap theater; it would mean the loss of advertising patronage; yet it is a fact that these theaters are the kindergartens of prostitution. Girls from 10 to 16 are admitted to witness plays which are essentially immoral. When the psychologic moment arrives,—as arrive it must to every female,—the result is either an immature prostitute or a sexual pervert. The police arrest the mature street walker, and she is fined or sent to prison. The child prostitute of the cheap theater is neglected and spreads disease broadcast. She is ignored and untreated,—a foul and pestilential creature hiding her nastiness under the cloak of childish innocence and youth. Society affects horror when such things are spoken of and hesitates to believe them.

The treatment the judiciary receives in this book is severe and justly so; yet why the author should express surprise at the rapidity of the trial and execution of Czolgosz is hard to say. The belief he gives expression to is "that if McKinley's assassin had been a wealthy man he would not have been tried and executed so quickly." One might pass over this with indulgence in view of the immense amount of good in the book if it were not a forecast of his very radical, almost dangerous, expressions on anarchy. Were it not for the balance of conscientious argument even though in spots it be illogic, Lydston's chapters on anarchy would certainly be alarming. A casual reader will form a wrong impression of his views in general and while we must agree with him that "instinctive anarchy (anarchy being here considered as rebellion against law and order) is a part of human psychology," one who has stood in the shadow of the red flag cannot possibly join him in expressions of tender solicitude for anarchy in general, nor add to his tears of regret at the untimely taking off of the Chicago anarchists.

It must be understood that Lydston in nowise sympathises with the law-breaker and the criminal; but he takes a wrong view of anarchy and most assuredly approaches perilously close to hero-worship in his defense of the memory of the men who died on the scaffold as a result of the Haymarket riot. He must be given credit for attempting to get at the truth of the affair; but he searches for it through eyes jaundiced by fixed ideas of misplaced sympathy. And, since his treatment of the anarchistic question is largely speculative and argumentative, the writer may be pardoned for referring back to the days of long ago. Lydston absolves the anarchist from evil tendencies and counts him the "under dog." He is a clever writer; he is brilliant, at times pyrotechnic, and he cuts corners beautifully; yet one whose experience with and knowledge of anarchy is rather more than casual must needs consider him if not actually biased, at least misled. For some time prior to the synchronous departure from this life of Spies, Parsons, Engel, and Fischer, the Chicago quartette, the writer was more or less intimately concerned in the study of anarchy in New York city as exemplified by that blatant, lump-jawed "patriot" John Most. He was intimately associated with the police in the surveillance of a destructionist group and knew each division of the anarchist world. When the Haymarket riot and bomb-throwing occurred the New York group was sought for opinions. All avowed anarchists denied knowledge of the Chicago crime; all deprecated it—in public; yet it was common gossip among the destructionists that a cipher telegram had brought the news of the explosion, glorifying the deed and deifying the doers. Lydston suggests a paranoic as the bomb thrower,—an independent worker in anarchy;—and says that of the men condemned Louis Ling was the only one of a type from whom such a deed might be expected. That is true. The others were of the higher type, the instigators, the generals.

That was rather conclusively, if bloodily, shown when Ling, in his cell, set off the explosive which created a vacancy where his face had been, a few hours before the time set for his execution. It showed too, that his intimate knowledge of explosives was less acute than his "patriotism." He was a poor, fear-bitten creature, cowering under the outspread wings of death, who lacked the stoicity of the martyr or the blind courage of the fanatic and fled from a fearsome end only to fling himself headlong into the ever-ready arms of the silent one.

It was the writer's privilege to witness the execution of the Chicago anarchists and he believed then from his intimate knowledge of the methods of the various groups that four very dangerous men were very properly, very completely and very justly hanged that morning in the Cook county jail. Time has not changed that opinion. The men executed may not have actually thrown the bomb; they probably did not; but they paved the way for the act and instigated it and were therefore guilty of the slaughter of men, for which they were hanged. Immediately after the execution in Chicago there was less red fire in New York.

As to Czolgosz, Lydston does not believe he was an anarchist, because he was repudiated by both the philosophic and destructive branches, which is the cut and dried method of the organisation whenever a removal is attempted or carried out, in proof of which we may refer to the denials of the New York group after the Chicago affair. Granting, for the sake of Lydston's argument, that he was not a regularly received and initiated anarchist, yet he avowed himself to be one; he was saturated with anarchistic ideas which he received from anarchistic literature, and he worked out his salvation according to the anarchistic doctrine. He was an anarchist of the purely destructive type. Viewed in a most liberal light anarchy, harmless as Lydston paints it, is nothing more than a benign tumor on the social body with a strong tendency to malignant degeneration. But mere nomenclature counts for little. The facts remain and point persistently to immigration as a source of evil and an etiologic factor in the causation of social disease. Lydston and the writer do not view anarchy from the same level, hence do not agree on some of the minor features of his chapter, which in nowise lessens the value of the book in the writer's opinion, although it mars a brilliant and learned work.

One feature of this work which appears to have been overlooked or ignored by reviewers is the chapter on the chemistry of social diseases. This view of the question is entirely original with Lydston and he is entitled to the fullest credit for bringing out a phase of a very important question which must in the future be considered with more than a mere passing interest. Its bearing on the cure of social diseases is vitally important; for, given a disease, its cure cannot be successfully brought about without a very complete and intelligent knowledge of the causes producing it. Lydston places toxemias and various poisons and their effects on the nervous system producing abnormal conditions at the head of the list, and then proceeds to elaborate autotoxemia as a factor in criminal etiology heretofore ignored.

Alcohol is placed in a unique position as "a disease producing vice and a vice producing disease." Its effects on various organs, the nervous system and on the natures of individuals is presented clearly, and the author suggests that every large city should be equipped with lock hospitals for the reception of alcoholics; that they should not be thrust in among the criminals. The relationship between toxemia and crime is established and there is food for deep thought in the connection between nerve tonics and patent medicines and degeneracy.

The volume is full of important and interesting statistics in relation to heredity and crime. Criminal families are traced out and the unbroken chain of degeneracy is displayed link by link. The negro question is interesting as Lydston presents it, although it will grate on some of the sentimentalists of the north. While he deprecates radical action he speaks a warning of the future. Quite interesting, too, is the author's explanation of the sexual perversions and criminal outbursts in men of middle and old age, who had hitherto borne excellent reputations and occupied high places in social and commercial life. Briefly, he calls it the climacteric in the male,—the psychic climacteric. This corroborates statements made in the genitourinary department of the JOURNAL, that the majority of men arrested for indecent exposures if examined would be found to be suffering from enlarged prostates.

There can be no question as to the value of the work in all it phases,—save only its defence of anarchy. It reflects the originality for which Lydston is famous and it is neither heavy, burdensome, nor dry. Written in his best style, it sparkles with epigrams, it cuts deep into social shams, and it is instructive. It should be widely read, not alone by scientific men but by the public, and especially that portion of it which dabbles in reform and charity—either as a pastime or a cloak. Beginning with social pathology the author considers the principles of evolution, the etiology, and the chemistry of social disease; sexual vice, and crime and their treatment; the race problem; genius and degeneracy, and the criminal's physical and psychic characteristics. The book is freely illustrated with excellent half tones of the crania and physiognomies of degenerates and criminals. Back of the work Lydston places a wide experience and a deep study of criminology and degeneracy; but at the expense of appearing hypercritical and forcing a purely personal opinion, 'twere better that the considerable portions of the anarchy chapters devoted to the Haymarket "innocents" and the "misguided" Czolgosz had been omitted. N. W. W.

